# Fungal Stress Database (FSD)––a repository of fungal stress physiological data

**DOI:** 10.1093/database/bay009

**Published:** 2018-02-12

**Authors:** Erzsébet Orosz, Nathalie van de Wiele, Tamás Emri, Miaomiao Zhou, Vincent Robert, Ronald P de Vries, István Pócsi

**Affiliations:** 1 Department of Biotechnology and Microbiology, Faculty of Science and Technology, University of Debrecen, Egyetem tér 1, H-4032 Debrecen, Hungary; 2 Fungal Physiology, Westerdijk Fungal Biodiversity Institute & Fungal Molecular Physiology, Utrecht University, Uppsalalaan 8, CT 3584 Utrecht, The Netherlands; 3 Bioinformatics Group, Westerdijk Fungal Biodiversity Institute, Uppsalalaan 8, CT 3584 Utrecht, The Netherlands

## Abstract

The construction of the Fungal Stress Database (FSD) was initiated and fueled by two major goals. At first, some outstandingly important groups of filamentous fungi including the aspergilli possess remarkable capabilities to adapt to a wide spectrum of environmental stress conditions but the underlying mechanisms of this stress tolerance have remained yet to be elucidated. Furthermore, the lack of any satisfactory interlaboratory standardization of stress assays, e.g*.* the widely used stress agar plate experiments, often hinders the direct comparison and discussion of stress physiological data gained for various fungal species by different research groups. In order to overcome these difficulties and to promote multilevel, e.g. combined comparative physiology-based and comparative genomics-based, stress research in filamentous fungi, we constructed FSD, which currently stores 1412 photos taken on *Aspergillus* colonies grown under precisely defined stress conditions. This study involved altogether 18 *Aspergillus* strains representing 17 species with two different strains for *Aspergillus niger* and covered six different stress conditions. Stress treatments were selected considering the frequency of various stress tolerance studies published in the last decade in the aspergilli and included oxidative (H_2_O_2_, menadione sodium bisulphite), high-osmolarity (NaCl, sorbitol), cell wall integrity (Congo Red) and heavy metal (CdCl_2_) stress exposures. In the future, we would like to expand this database to accommodate further fungal species and stress treatments.

URL: http://www.fung-stress.org/

## Introduction 

The number of the versatile species belonging to the monophyletic kingdom Fungi may exceed 5 million as demonstrated by high-throughput sequencing of environmental DNA samples (1, 2). The remarkable success and diversity of today’s fungi can be attributed, at least in part, to their outstanding armory evolved to combat various types of environmental stress (3). Shedding new light on the elements of the stress sensing, signaling and stress response systems operating in fungi may provide us with indispensable tools to control fungal growth in a wide spectrum of ecological niches, which are occupied frequently and successfully by either saprophytic or parasitic fungi (3). In addition, modulation of elements of the fungal stress adaptation systems may also lead to the development of new stress-tolerant industrial strains for a number of various biotechnological applications (3–5). Not surprisingly, the number of publications concerning the stress biology of fungi increases steadily and approached as many as 700 per year in 2011 (3). In addition, a new scientific meeting was born in 2014 about fungal stress that was called ‘International Symposium on Fungal Stress––ISFUS’ (6, 7) and the second meeting was held in Goiânia (Brazil) in 2017 (https://isfus.wordpress.com/) (8). The logo of the International Symposium on Fungal Stress–ISFUS features one of the most-studied ascomycetes, *Aspergillus nidulans* and illustrates several key stress parameters that fungi cope with to survive.

**Figure 1.**
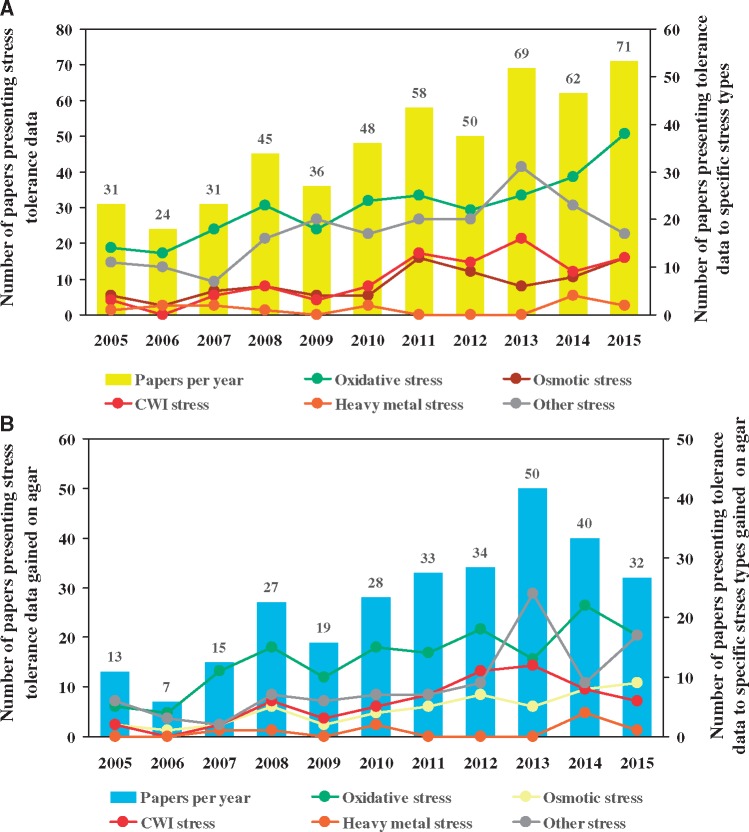
Scientific publications reporting on stress experiments performed in the aspergilli between 2005 and 2015. Columns represent the annual number of papers relying on stress tolerance data gained in any kind of stress assays (**A**) or stress tolerance data gained in stress agar experiments only (**B**). Closed symbols connected by lines show the annual number of papers presenting tolerance data to specific stress types in general (A) or data to specific stress types only gained on stress agar plates (B). More information on the annual numbers of stress-related papers published in fungi between 2000 and 2011 is available in the previous publication by Karányi *et al.* (3).

Among fungi, ascomycetous species and especially those belonging to the genus *Aspergillus* have attracted enormous professional and even public attention. It is noteworthy that the responsiveness of some important developmental processes (sporulation, biofilm formation) and metabolic pathways (e.g. mycotoxin production) to environmental (predominantly oxidative) stress has been demonstrated in the aspergilli (4, 9–12). According to a literature search carried out by us in the Web of Science^®^ database [Advanced search: TS=(stress) AND TI=(*Aspergillus*) indexes = SCI-EXPANDED, SSCI, A&HCI, ESCI Timespan = 2005–15], 525 stress-related papers were published on the aspergilli between 2005 and 2015 (Figure 1, Supplementary Table S1). Importantly, 56.8% of the publications (298) reported on data gained in stress agar experiments as found by manual search of the literature available in the Web of Science^®^ database, and the total number of stress conditions tested in these assays was 362 (Figure 1, Supplementary Table S2). The distribution of stress agar data among major stress types were uneven with the predominance of oxidative stress assays (144 tests, 39.8% of total), and with a sensibly less interest in osmotic (50 assays, 13.8%), cell wall integrity (CWI; 62 assays, 17.1%) and heavy metal (9 assays, 2.5%) stress agar studies (Figure 1, Supplementary Table S2).

Stress agar experiments have also been carried out by us as part of the daily routine in our laboratory, especially with gene deletion and gene over-expression strains of *A. nidulans* (4, 5, 13–18). Unfortunately, the interlaboratory standardization of such stress agar assays has remained to be done, even though the standardization of these widely used assays seems unavoidable. Variations in the culture conditions, e.g. addition of auxotrophy supplements to the culture media even at low concentrations (13), changes in the incubation temperature of the cultures (19) or alterations in the production of conidiospores for the inoculation of the stress agar plates (20, 21) may influence the outcomes of these stress agar experiments. This means that even slight differences in the protocols of the stress agar plate assays may prevent us to make any direct comparison and any joint discussion of stress tolerance data gained by different fungal laboratories for various fungal species.

Therefore, we were highly motivated to set up the Fungal Stress Database (FSD; http://www.fung-stress.org/), where pictures of stress agar plate cultures performed with selected *Aspergillus* species have been made available to the public for the comparison of stress tolerance data gained in various laboratories and also for further analyses. All pictures are supplied with a full description of the culture conditions including sporulation conditions, stressor concentrations as well as incubation times and temperatures. Most recently, the stress physiological dataset presented in FSD has been used successfully by de Vries *et al.* (18) to find causal connections between the varying stress tolerances of the aspergilli and the genome-level differences in their stress defense gene sets.

## Construction

**Figure 2.**
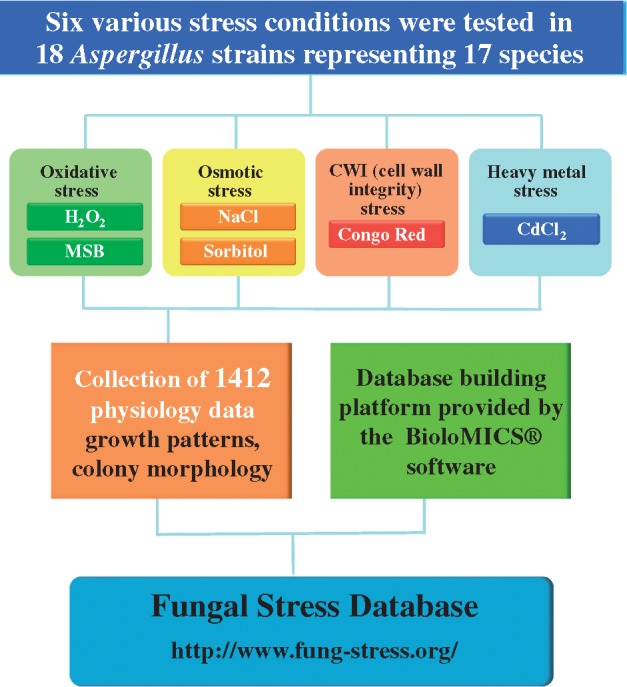
Workflow of the construction of the FSD.

The workflow of the construction of FSD is presented in Figure 2. Altogether 18 *Aspergillus* strains representing 17 species {*A. aculeatus* (CBS 172.66), *A. brasiliensis* (CBS 101740), *A. carbonarius* (CBS 141172 = DTO 115-B6), *A. clavatus* (CBS 513.65 = NRRL1), *A. fischeri* (CBS 544.65), *A. flavus* (CBS 128202 = NRRL 3357), *A. fumigatus* (CBS 126847 = Af293), *A. glaucus* (CBS 516.65), *A. luchuensis* (CBS 106.47), *A. nidulans* (FGSCA4), *Aspergillusniger* represented by two strains (CBS 113.46 and N402), *A. oryzae* (Rib40), *A. sydowii* (CBS 593.65), *A. terreus* (NIH2624), *A. tubingensis* (CBS 134.48), *A. versicolor* (CBS 795.97), *A. wentii* (CBS 141173 = DTO 134-E9)} were exposed to six well-defined stress conditions representing oxidative (H_2_O_2_, menadione sodium bisulfite), osmotic (NaCl, sorbitol), CWI (Congo Red) and heavy metal (CdCl_2_) stress. Stress conditions were selected considering the frequency of the use of different stress agar assays for various stress types in the literature between 2005 and 2015 (Figure 1, Supplementary Table S2). Importantly, approximately three quarters (73.2%) of the *Aspergillus* stress biology papers published in this time period reported on the results of stress agar experiments performed under oxidative, osmotic, CWI and/or heavy metal stress conditions (Figure 1, Supplementary Table S2). Finally, altogether 1412 photos taken on *Aspergillus* colony growth and morphology were collected and placed in FSD (http://www.fung-stress.org/; Westerdijk Fungal Biodiversity Institute, Utrecht) (Figure 2).

A detailed description of the stress physiology experiments on nutrient stress agar plates is available at the FSD web site (click on ‘GROWTH MEDIA’ and choose the link shown under ‘Experimental conditions:’ or, alternatively, download http://www.fung-stress.org/Files/ExperimentalConditionsDetailed.pdf) and also in the paper of de Vries *et al.* (18). Briefly, conidia for inoculation of the agar plates were produced typically on malt extract––mycological pepton sporulation agar medium (1.5% agar, 25°C in the dark, 6 days; http://www.fung-stress.org/) (18). Importantly, the sporulation medium for the osmophilic *A. glaucus* species was always supplemented with 1.0 M NaCl and, in selected experiments, *A. fumigatus* and *A. nidulans* were also sporulated at 37°C on either the same malt extract—mycological pepton—agar sporulation medium or, alternatively, on nitrate minimal medium agar plates (NMM with 1.8% agar) (22). Stress agar experiments were performed on NMM agar plates, which were supplemented with stress-initiating agents as required and were incubated at 25 (5 or 10 days) or 37°C (5 days) in the dark. Importantly, stress initiating agents were also supplemented at 55°C just above the rigidification temperature of agar by adding pre-calculated volumes of stock solutions. The point inoculation of stress agar plates (with 10^5^ freshly grown conidia washed and suspended in 5 μl 0.9% NaCl, 0.01% Tween 80), monitoring the growth of the colonies, archiving and evaluating colony pictures as well as quantifying stress-elicited growth inhibitions were carried out as described in details in previous publications (13, 14, 16, 18). The growth of the colonies was always characterized by colony diameters (in cm) (16) and also by scoring them by eye (on a 0–10 scale) (18).

FSD and the FSD website are based on the BioloMICS software (23), which is a tool allowing specialized and scientific biological databases to be created to fit the specific needs of researchers working on any organisms. The system is flexible enough to accommodate any type of data and new fields can be created on the fly without the intervention of software developers. Data management is made simple for curators using the desktop version of the software and a number of tools for data retrieval and analysis are also included (https://www.bio-aware.com/Defaultinfo.aspx? Page=BioloMICSNet). The FSD website is based on the online version of the same software (https://www.bio-aware.com/Defaultinfo.aspx? Page=BioloMICSWeb).

## Database interface and visualization

**Figure 3.**
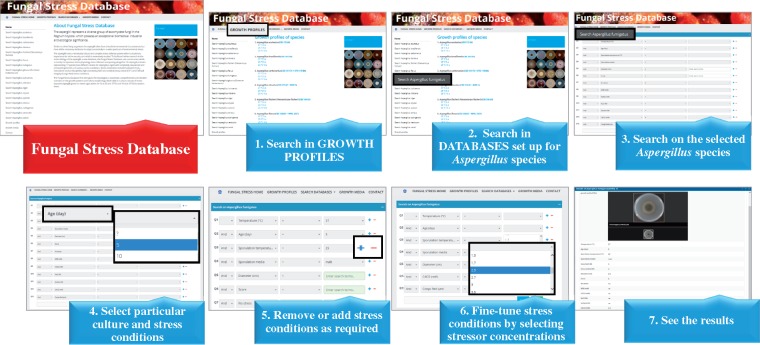
Search in the FSD.

The FSD interface (http://www.fung-stress.org/) and a short presentation of search in FSD are shown in Figure 3. A typical search starts with clicking on ‘GROWTH PROFILES’ and follows with the selection of species, incubation temperature and incubation time (age of culture). All pictures taken for one species at a given incubation temperature and time are collected in PDF files, which are freely available for the users after clicking on one of the three optional combinations of the culture conditions listed under the species names: ‘37°C 5 d’, ‘25°C 5 d’ or ‘25°C 10 d.’ The visitors are advised to down-load and save/print out the PDF files of their interest. After then, they may enter ‘SEARCH DATABASES,’ select species, incubation temperature (25 or 37°C), age of culture (5 or 10 d), sporulation temperature (25 or 37°C, only for *A. fumigatus* and *A. nidulans*) and media (malt extract or NMM, only for *A. fumigatus* and *A. nidulans*), the inclusion of extra NaCl (*A. glaucus*) or extra sorbitol (*A. glaucus* and *A. wentti*) in the culture media as well as ‘No stress’ condition or MSB, H_2_O_2_, NaCl, sorbitol, CdCl_2_ and Congo Red elicited stresses. Considering actual stressor concentrations, we suggest that these values should be chosen from the data shown in the PDF files having been gained under ‘GROWTH PROFILES’ to make any search in FSD time-saving and more efficient. Choosing the ‘GROWTH PROFILES’ menu option, the users can also gain an access to links to the descriptions of the fungal strains used in this study (‘CBS strain database’; http://www.fung-stress.org/DefaultInfo.aspx? Page=Species).

Pictures of stress agar cultures obeying the actual search conditions will appear after clicking on ‘Search’ and the growth profiles of the colonies with full descriptions of culture conditions including incubation temperature, age of the colony, extra NaCl (optional, only for *A. glaucus*), extra sorbitol (optional, only for *A. glaucus* and *A. wentii*), colony diameters (in cm and on a 0–10 scale), name as well as concentration of the stressor added to the culture media, sporulation temperature (optional, only for *A. fumigatus* and *A. nidulans*) and sporulation media (optional, only for *A. fumigatus* and *A. nidulans*) can be assessed easily on the screen. New search with the same species can be started comfortably by clicking on ‘Reset.’

Colonies can also be sorted by setting arbitrary upper limits on their Diameter (calculated in cm) or Score values. If somebody is interested in no growth or negligible growth conditions setting Diameter (cm) to < 1 cm or Score to < 1 can be effective. It is worth noting that when any query is processed for *A. niger* two sets of pictures will always appear for the strains *A. niger* CBS 113.46 and NCCB 402, which will make any direct comparison of the stress tolerances of these strains quite easy.

The FSD interface (http://www.fung-stress.org/) also provides the researchers with the links to ‘FUNGAL STRESS HOME,’ ‘GROWTH MEDIA’ and ‘CONTACT.’ Names and availabilities of the responsible persons for data related matters (RPV, IP and EO) and for software and website (VR) can be found under ‘FUNGAL STRESS HOME.’ You may also find here shortcuts to ‘Westerdijk Institute Home,’ ‘Fungal Growth Database’ and ‘Fungal Stress Response Database.’ To facilitate the reproduction of the growth profiles, a full description of all culture media used in the construction of FSD is available under ‘GROWTH MEDIA.’ Finally, you can leave an e-mail message to any co-authors of present paper under ‘CONTACT.’

## Conclusion

Stress responses and stress-related physiological and developmental processes are important but still understudied areas in fungal biology. The number of papers aiming at the deciphering of the remarkable stress tolerance of fungi is increasing steadily. To stimulate further research in the field of fungal stress biology and to facilitate future interlaboratory standardizations of agar stress plate assays, the FSD was constructed, which currently stores almost 1.5 thousand photos on various *Aspergillus* colonies grown on well-defined stress agar media. A detailed description of the culture media and the tested stress conditions may help researchers to reproduce these data easily and use them in their stress biology studies as basic references. This version of FSD we presented here may serve as the beginning of the discussion within the community of fungal stress biology experts to evaluate the current situation, which will hopefully lead to further development in standardization. In addition, the FSD dataset may also provide us with good-quality physiological data for combined comparative stress-physiology and comparative genomics projects (18). Finally, we hope that FSD will also incorporate stress tolerance data for various fungal species other than the aspergilli in the near future.

## Supplementary data

Supplementary data are available at *Database* Online.

## Supplementary Material

Supplementary TableS1

Supplementary TableS2

## References

[1] O’Brien H.E., Parrent J.L., Jackson J.A. (2005) Fungal community analysis by large-scale sequencing of environmental samples. Appl. Environ. Microbiol., 71, 5544–5550. 16151147 10.1128/AEM.71.9.5544-5550.2005 PMC1214672

[2] Blackwell M. (2011) The fungi: 1, 2, 3 … 5.1 million species? Am. J. Bot., 98, 426–438. 21613136 10.3732/ajb.1000298

[3] Karányi Z., Holb I., Hornok L. (2013) FSRD: fungal stress response database. Database (Oxford), 2013, bat037. 23757396 10.1093/database/bat037 PMC3678302

[4] Emri T., Szarvas V., Orosz E. (2015) Core oxidative stress response in *Aspergillus nidulans*. BMC Genomics, 16, 478. 26115917 10.1186/s12864-015-1705-z PMC4482186

[5] Leiter É., Park H.S., Kwon N.J. (2016) Characterization of the *aodA*, *dnmA*, *mnSOD* and *pimA* genes in *Aspergillus nidulans*. Sci. Rep., 6, 20523. 26846452 10.1038/srep20523 PMC4742808

[6] Rangel D.E.N., Alder-Rangel A., Dadachova E. (2015a) Fungal stress biology: a preface to the Fungal Stress Responses special edition. Curr. Genet., 61, 231–238. 26116075 10.1007/s00294-015-0500-3

[7] Rangel D.E.N., Alder-Rangel A., Dadachova E. (2015) The International symposium on fungal stress: iSFUS. Curr. Genet., 61, 479–487. 26100601 10.1007/s00294-015-0501-2

[8] Alder-Rangel A., Bailao A.M., da Cunha A.F. (2017) *The Second International Symposium on Fungal Stress: ISFUS. Fungal Biol.*, 10.1016/j.funbio.2017.10.011. 29801782

[9] Reverberi M., Ricelli A., Zjalic S. (2010) Natural functions of mycotoxins and control of their biosynthesis in fungi. Appl. Microbiol. Biotechnol., 87, 899–911. 20495914 10.1007/s00253-010-2657-5

[10] Hong S.Y., Roze L.V., Linz J.E. (2013) Oxidative stress-related transcription factors in the regulation of secondary metabolism. Toxins (Basel), 5, 683–702. 23598564 10.3390/toxins5040683 PMC3705287

[11] Jaimes-Arroyo R., Lara-Rojas F., Bayram Ö. (2015) The SrkA kinase is part of the SakA mitogen-activated protein kinase interactome and regulates stress responses and development in *Aspergillus nidulans*. Eukaryot. Cell, 14, 495–510. 25820520 10.1128/EC.00277-14 PMC4421008

[12] Zheng H., Kim J., Liew M. (2015) Redox metabolites signal polymicrobial biofilm development via the NapA oxidative stress cascade in *Aspergillus*. Curr. Biol., 25, 29–37. 25532893 10.1016/j.cub.2014.11.018 PMC4286458

[13] Balázs A., Pócsi I., Hamari Z. (2010) AtfA bZIP-type transcription factor regulates oxidative and osmotic stress responses in *Aspergillus nidulans*. Mol. Genet. Genomics, 283, 289–303. 20131067 10.1007/s00438-010-0513-z

[14] Leiter É., González A., Erdei É. (2012) Protein phosphatase Z modulates oxidative stress response in fungi. Fungal Genet. Biol., 49, 708–716. 22750657 10.1016/j.fgb.2012.06.010

[15] Kovács Z., Szarka M., Kovács S. (2013) Effect of cell wall integrity stress and RlmA transcription factor on asexual development and autolysis in *Aspergillus nidulans*. Fungal Genet. Biol., 54, 1–14. 23485399 10.1016/j.fgb.2013.02.004

[16] Yin W.B., Reinke A.W., Szilágyi M. (2013) bZIP transcription factors affecting secondary metabolism, sexual development and stress responses in *Aspergillus nidulans*. Microbiology, 159, 77–88. 23154967 10.1099/mic.0.063370-0 PMC3542729

[17] Leiter É., Bálint M., Miskei M. (2016b) Stress tolerances of nullmutants of function-unknown genes encoding menadione stress-responsive proteins in *Aspergillus nidulans*. J. Basic Microbiol., 56, 827–833. 26631869 10.1002/jobm.201500500

[18] de Vries R.P., Riley R., Wiebenga A. (2017) Comparative genomics reveals high biological diversity and specific adaptation in the industrially and medically important fungal genus *Aspergillus*. Genome Biol., 18, 28. 28196534 10.1186/s13059-017-1151-0 PMC5307856

[19] Han K.H., Prade R.A. (2002) Osmotic stress-coupled maintenance of polar growth in *Aspergillus nidulans*. Mol. Microbiol., 43, 1065–1078. 11918796 10.1046/j.1365-2958.2002.02774.x

[20] Nanguy S.P., Perrier-Cornet J.M., Bensoussan M. (2010) Impact of water activity of diverse media on spore germination of *Aspergillus* and *Penicillium* species. Int. J. Food Microbiol., 142, 273–276. 20673593 10.1016/j.ijfoodmicro.2010.06.031

[21] Nguyen Van Long N., Vasseur V., Coroller L. (2017) Temperature, water activity and pH during conidia production affect the physiological state and germination time of *Penicillium* species. Int. J. Food Microbiol., 241, 151–160. 27780083 10.1016/j.ijfoodmicro.2016.10.022

[22] Barratt R.W., Johnson G.B., Ogata W.N. (1965) Wild-type and mutant stocks of *Aspergillus nidulans*. Genetics, 52, 233–246. 5857598 10.1093/genetics/52.1.233 PMC1210840

[23] Robert V., Szoke S., Jabas B. (2011) BioloMICS software: biological data management, identification, classification and statistics. Open Appl. Inform. J., 5, 87–98.

