# Supplementary material for: Fungal Stress Database (FSD)––a repository of fungal stress physiological data
Source: Database (Oxford). 2018 Feb 12;2018:bay009. doi: 10.1093/database/bay009 (PMC5810435; doi:10.1093/database/bay009)
Supplement: Supplementary TableS2 [file bay009_supp_tables2.doc]

**Supplementary Table S2.** Publications in the aspergilli involving stress agar plate experiments between 2005-2015

| **Summary Table** | | | | | | | |
| --- | --- | --- | --- | --- | --- | --- | --- |
| **Year** | **Number of papers** | **Stress types** | | | | | **The total number of stress conditions tested in the papers** |
|  |  | **Oxidative** | **Osmotic** | **CWI** | **Heavy metal** | **Other** |  |
| 2005 | **13** | 5 | 2 | 2 | 0 | 6 | **15** |
| 2006 | **7** | 4 | 1 | 0 | 0 | 3 | **8** |
| 2007 | **15** | 11 | 2 | 2 | 1 | 2 | **18** |
| 2008 | **27** | 15 | 5 | 6 | 1 | 7 | **34** |
| 2009 | **19** | 10 | 2 | 3 | 0 | 6 | **21** |
| 2010 | **28** | 15 | 4 | 5 | 2 | 7 | **33** |
| 2011 | **33** | 14 | 5 | 7 | 0 | 7 | **33** |
| 2012 | **34** | 18 | 7 | 11 | 0 | 9 | **45** |
| 2013 | **50** | 13 | 5 | 12 | 0 | 24 | **54** |
| 2014 | **40** | 22 | 8 | 8 | 4 | 9 | **51** |
| 2015 | **32** | 17 | 9 | 6 | 1 | 17 | **50** |
| **Sum** | **298** | **144** | **50** | **62** | **9** | **97** | **362** |
|  |  | **362** | | | | |  |

| **Publications in the aspergilli involving stress agar plate experiments between 2005-2015** | | | **PubMed/DOI/ISSN IDs** | **Stress types** | | |
| --- | --- | --- | --- | --- | --- | --- |
| **Year** | **Papers** | |  |  |  |  |
| **2015** | **1** | **Title:**A Caleosin-Like Protein with Peroxygenase Activity Mediates Aspergillus flavus Development, Aflatoxin Accumulation, and Seed Infection | **PubMed ID:**26116672 | **oxidative** |  |  |
|  | **2** | **Title:**The Aspergillus fumigatus pkcA(G579R) Mutant Is Defective in the Activation of the Cell Wall Integrity Pathway but Is Dispensable for Virulence in a Neutropenic Mouse Infection Model | **PubMed ID:**26295576 | **oxidative** |  |  |
|  | **3** | **Title:**The Aspergillus fumigatus sitA Phosphatase Homologue Is Important for Adhesion, Cell Wall Integrity, Biofilm Formation, and Virulence | **PubMed ID:**25911225 | **oxidative** | **CWI** | **heavy metal** |
|  | **4** | **Title:**Systematic Global Analysis of Genes Encoding Protein Phosphatases in Aspergillus fumigatus | **PubMed ID:**25943523 | **oxidative** | **CWI** |  |
|  | **5** | **Title:**Mitogen-activated protein kinases MpkA and MpkB independently affect micafungin sensitivity in Aspergillus nidulans | **PubMed ID:**25727969 | **oxidative** | **CWI** |  |
|  | **6** | **Title:**The SrkA Kinase Is Part of the SakA Mitogen-Activated Protein Kinase Interactome and Regulates Stress Responses and Development in Aspergillus nidulans | **PubMed ID:**25820520 | **oxidative** | **osmotic** |  |
|  | **7** | **Title:**KdmA, a histone H3 demethylase with bipartite function, differentially regulates primary and secondary metabolism in Aspergillus nidulans | **PubMed ID:**25712266 | **oxidative** |  |  |
|  | **8** | **Title:**The Aspergillus fumigatus cell wall integrity signaling pathway: drug target, compensatory pathways, and virulence | **PubMed ID:**25932027 | **oxidative** | **CWI** |  |
|  | **9** | **Title:**Inhibition of aflatoxin metabolism and growth of Aspergillus flavus in liquid culture by a DNA methylation inhibitor | **PubMed ID:**25312249 | **oxidative** |  |  |
|  | **10** | **Title:**Mechanism of Cr(VI) reduction by Aspergillus niger: enzymatic characteristic, oxidative stress response, and reduction product | **PubMed ID:**25408081 | **oxidative** | **CWI** |  |
|  | **11** | **Title:**Identification and mutational analyses of phosphorylation sites of the calcineurin-binding protein CbpA and the identification of domains required for calcineurin binding in Aspergillus fumigatus | **PubMed ID:**25821446 | **oxidative** |  |  |
|  | **12** | **Title:**Morphological transitions under oxidative stress in response to metabolite formation in Aspergillus niger | **PubMed ID:**25367801 | **oxidative** | **osmotic** |  |
|  | **13** | **Title:**Redox Metabolites Signal Polymicrobial Biofilm Development via the NapA Oxidative Stress Cascade in Aspergillus | **PubMed ID:**25532893 | **oxidative** |  |  |
|  | **14** | **Title:**Aspergillus flavus impairs antioxidative enzymes of Sternochetus mangzferae during mycosis | **PubMed ID:**25446036 | **oxidative** |  |  |
|  | **15** | **Title:**Reactive oxygen species regulate lovastatin biosynthesis in Aspergillus terreus during submerged and solid-state fermentations | **PubMed ID:**25457945 | **oxidative** |  |  |
|  | **16** | **Title:**The role of AtfA and HOG MAPK pathway in stress tolerance in conidia of Aspergillus fumigatus | **PubMed ID:**25459537 | **oxidative** | **osmotic** |  |
|  | **17** | **Title:**Whole-Genome Comparison of Aspergillus fumigatus Strains Serially Isolated from Patients with Aspergillosis | **PubMed ID:**25232160 | **oxidative** |  |  |
|  | **18** | **Title:**Network Modeling Reveals Cross Talk of MAP Kinases during Adaptation to Caspofungin Stress in Aspergillus fumigatus | **PubMed ID:**26356475 | **osmotic** |  |  |
|  | **19** | **Title:**Concomitant osmotic and chaotropicity-induced stresses in Aspergillus wentii: compatible solutes determine the biotic window | **PubMed ID:**26055444 | **osmotic** |  |  |
|  | **20** | **Title:**Proteomic profile of Aspergillus flavus in response to water activity | **PubMed ID:**25749363 | **osmotic** |  |  |
|  | **21** | **Title:**A Ribosomal Protein AgRPS3aE from Halophilic Aspergillus glaucus Confers Salt Tolerance in Heterologous Organisms | **PubMed ID:**25642759 | **osmotic** |  |  |
|  | **22** | **Title:**Negative regulation of the vacuole-mediated resistance to K+ stress by a novel C2H2 zinc finger transcription factor encoded by aslA in Aspergillus nidulans | **PubMed ID:**25626364 | **osmotic** |  |  |
|  | **23** | **Title:**Expression, characterization and mutagenesis of an FAD-dependent glucose dehydrogenase from Aspergillus terreus | **PubMed ID:**25435504 | **osmotic** |  |  |
|  | **24** | **Title:**The Aspergillus fumigatus septins play pleiotropic roles in septation, conidiation, and cell wall stress, but are dispensable for virulence | **PubMed ID:**26051489 | **CWI** |  |  |
|  | **25** | **Title:**Kexin-like endoprotease KexB is required for N-glycan processing, morphogenesis and virulence in Aspergillus fumigatus | **PubMed ID:**25687931 | **CWI** |  |  |
|  | **26** | **Title:**Protein kinase C regulates the expression of cell wall-related genes in RlmA-dependent and independent manners in Aspergillus nidulans | **PubMed ID:**25345444 | **CWI** |  |  |
|  | **27** | **Title:**Hsp70 and the Cochaperone StiA (Hop) Orchestrate Hsp90-Mediated Caspofungin Tolerance in Aspergillus fumigatus | **PubMed ID:**26014950 | **other** |  |  |
|  | **28** | **Title:**Calcium signaling mediates antifungal activity of triazole drugs in the Aspergilli | **PubMed ID:**25554700 | **other** |  |  |
|  | **29** | **Title:**Structural analysis of novel trehalose-based oligosaccharides from extremely stress-tolerant ascospores of Neosartorya fischeri (Aspergillus fischeri) | **PubMed ID:**25974853 | **other** |  |  |
|  | **30** | **Title:**A second component of the SltA-dependent cation tolerance pathway in Aspergillus nidulans | **PubMed ID:**26119498 | **other** |  |  |
|  | **31** | **Title:**Switching from a Unicellular to Multicellular Organization in an Aspergillus niger Hypha | **PubMed ID:**25736883 | **other** |  |  |
|  | **32** | **Title:**Identification of functional cis-elements required for repression of the Taka-amylase A gene under secretion stress in Aspergillus oryzae | **PubMed ID:**25280730 | **other** |  |  |
| **2014** | **33** | **Title:**Illumina identification of RsrA, a conserved C2H2 transcription factor coordinating the NapA mediated oxidative stress signaling pathway in Aspergillus | **PubMed ID:**25416206 | **oxidative** | **heavy metal** |  |
|  | **34** | **Title:**ChIP-seq reveals a role for CrzA in the Aspergillus fumigatus high-osmolarity glycerol response (HOG) signalling pathway | **PubMed ID:**25196896 | **oxidative** | **osmotic** |  |
|  | **35** | **Title:**Perturbations in small molecule synthesis uncovers an iron-responsive secondary metabolite network in Aspergillus fumigatus | **PubMed ID:**25386169 | **oxidative** |  |  |
|  | **36** | **Title:**Buckwheat achenes antioxidant profile modulates Aspergillus flavus growth and aflatoxin production | **PubMed ID:**25108759 | **oxidative** |  |  |
|  | **37** | **Title:**A dually located multi-HMG-box protein of Aspergillus nidulans has a crucial role in conidial and ascospore germination | **PubMed ID:**25156107 | **oxidative** |  |  |
|  | **38** | **Title:**Interplay between proteases and protease inhibitors in the sea fan-Aspergillus pathosystem | **DOI:**10.1007/s00227-014-2499-2 | **oxidative** |  |  |
|  | **39** | **Title:**Characterization of the Aspergillus fumigatus detoxification systems for reactive nitrogen intermediates and their impact on virulence | **PubMed ID:**25309516 | **oxidative** |  |  |
|  | **40** | **Title:**A Proteomic Approach to Investigating Gene Cluster Expression and Secondary Metabolite Functionality in Aspergillus fumigatus | **PubMed ID:**25198175 | **oxidative** | **heavy metal** |  |
|  | **41** | **Title:**Aflatoxin inhibition in Aspergillus flavus for bioremediation purposes | **DOI:**10.1007/s13213-013-0732-8 | **oxidative** | **osmotic** |  |
|  | **42** | **Title:**An Antifungal Role of Hydrogen Sulfide on the Postharvest Pathogens Aspergillus niger and Penicillium italicum | **PubMed ID:**25101960 | **oxidative** |  |  |
|  | **43** | **Title:**Halophilic Aspergillus penicillioides from athalassohaline, thalassohaline, and polyhaline environments | **PubMed ID:**25140168 | **oxidative** | **CWI** |  |
|  | **44** | **Title:**VeA Is Associated with the Response to Oxidative Stress in the Aflatoxin Producer Aspergillus flavus | **PubMed ID:**24951443 | **oxidative** | **osmotic** |  |
|  | **45** | **Title:**The Involvement of the Mid1/Cch1/Yvc1 Calcium Channels in Aspergillus fumigatus Virulence | **PubMed ID:**25083783 | **oxidative** | **CWI** |  |
|  | **46** | **Title:**Effect of low shear modeled microgravity on phenotypic and central chitin metabolism in the filamentous fungi Aspergillus niger and Penicillium chrysogenum | **PubMed ID:**24803238 | **oxidative** | **CWI** |  |
|  | **47** | **Title:**Cadmium-induced oxidative stress tolerance in cadmium resistant Aspergillus foetidus: its possible role in cadmium bioremediation | **PubMed ID:**24836877 | **oxidative** | **heavy metal** |  |
|  | **48** | **Title:**The loss of the inducible Aspergillus carbonarius MFS transporter MfsA leads to ochratoxin A overproduction | **PubMed ID:**24791702 | **oxidative** |  |  |
|  | **49** | **Title:**Temperature-stress tolerance of the fungal strain Aspergillus niger 26: physiological and ultrastructural changes | **PubMed ID:**24366816 | **oxidative** |  |  |
|  | **50** | **Title:**The non-metabolizable glucose analog D-glucal inhibits aflatoxin biosynthesis and promotes kojic acid production in Aspergillus flavus | **PubMed ID:**24742119 | **oxidative** |  |  |
|  | **51** | **Title:**Effects of Ozone Gas Exposure on Toxigenic Fungi Species from Fusarium, Aspergillus, and Penicillium Genera | **DOI:**10.1080/01919512.2013.846824 | **oxidative** |  |  |
|  | **52** | **Title:**Environmental influences on maize-Aspergillus flavus interactions and aflatoxin production | **PubMed ID:**24550905 | **oxidative** |  |  |
|  | **53** | **Title:**Linkage of Oxidative Stress and Mitochondrial Dysfunctions to Spontaneous Culture Degeneration in Aspergillus nidulans | **PubMed ID:**24345786 | **oxidative** |  |  |
|  | **54** | **Title:**The Aspergillus nidulans ATM Kinase Regulates Mitochondrial Function, Glucose Uptake and the Carbon Starvation Response | **PubMed ID:**24192833 | **oxidative** |  |  |
|  | **55** | **Title:**Global Survey of Canonical Aspergillus flavus G Protein-Coupled Receptors | **PubMed ID:**25316696 | **osmotic** |  |  |
|  | **56** | **Title:**Functional analysis of histone deacetylase and its role in stress response, drug resistance and solid-state cultivation in Aspergillus oryzae | **PubMed ID:**24613105 | **osmotic** | **CWI** |  |
|  | **57** | **Title:**Abiotic Stress Resistance, a Novel Moonlighting Function of Ribosomal Protein RPL44 in the Halophilic Fungus Aspergillus glaucus | **PubMed ID:**24814782 | **osmotic** | **heavy metal** |  |
|  | **58** | **Title:**Regulatory mutations affecting sulfur metabolism induce environmental stress response in Aspergillus nidulans | **PubMed ID:**24513272 | **osmotic** |  |  |
|  | **59** | **Title:**Distinct Septin Heteropolymers Co-Exist during Multicellular Development in the Filamentous Fungus Aspergillus nidulans | **PubMed ID:**24664283 | **osmotic** |  |  |
|  | **60** | **Title:**Protein Kinase C Overexpression Suppresses Calcineurin-Associated Defects in Aspergillus nidulans and Is Involved in Mitochondrial Function | **PubMed ID:**25153325 | **CWI** |  |  |
|  | **61** | **Title:**The Aspergillus fumigatus beta-1,3-glucanosyltransferase Gel7 plays a compensatory role in maintaining cell wall integrity under stress conditions | **PubMed ID:**24429506 | **CWI** |  |  |
|  | **62** | **Title:**Functional analysis of the C-II subgroup killer toxin-like chitinases in the filamentous ascomycete Aspergillus nidulans | **PubMed ID:**24384382 | **CWI** |  |  |
|  | **63** | **Title:**Polysome profiling reveals broad translatome remodeling during endoplasmic reticulum (ER) stress in the pathogenic fungus Aspergillus fumigatus | **PubMed ID:**24568630 | **CWI** |  |  |
|  | **64** | **Title:**Two C4-sterol methyl oxidases (Erg25) catalyse ergosterol intermediate demethylation and impact environmental stress adaptation in Aspergillus fumigatus | **PubMed ID:**25107308 | **other** |  |  |
|  | **65** | **Title:**Production of Kluyveromyces spp. and environmental tolerance induction against Aspergillus flavus | **DOI:**10.1007/s13213-013-0726-6 | **other** |  |  |
|  | **66** | **Title:**Aspergillus flavus infection induces transcriptional and physical changes in developing maize kernels | **PubMed ID:**25132833 | **other** |  |  |
|  | **67** | **Title:**Genetically shaping morphology of the filamentous fungus Aspergillus glaucus for production of antitumor polyketide aspergiolide A | **PubMed ID:**24886193 | **other** |  |  |
|  | **68** | **Title:**Trehalose synthesis in Aspergillus niger: characterization of six homologous genes, all with conserved orthologs in related species | **PubMed ID:**24725382 | **other** |  |  |
|  | **69** | **Title:**Identification of a Key Lysine Residue in Heat Shock Protein 90 Required for Azole and Echinocandin Resistance in Aspergillus fumigatus | **PubMed ID:**24395240 | **other** |  |  |
|  | **70** | **Title:**Distribution, expression and expansion of Aspergillus fumigatus LINE-like retrotransposon populations in clinical and environmental isolates | **PubMed ID:**24440682 | **other** |  |  |
|  | **71** | **Title:**Aspergillus penicillioides-a true halophile existing in hypersaline and polyhaline econiches | **DOI:**10.1007/s13213-013-0646-5 | **other** |  |  |
|  | **72** | **Title:**Dissimilatory nitrate reduction by Aspergillus terreus isolated from the seasonal oxygen minimum zone in the Arabian Sea | **PubMed ID:**24517718 | **other** |  |  |
| **2013** | **73** | **Title:**Deletion of the putative stretch-activated ion channel Mid1 is hypervirulent in Aspergillus fumigatus | **PubMed ID:**24239700 | **oxidative** | **osmotic** | **CWI** |
|  | **74** | **Title:**Increase of Fumonisin B-2 and Ochratoxin A Production by Black Aspergillus Species and Oxidative Stress in Grape Berries Damaged by Powdery Mildew | **PubMed ID:**24290677 | **oxidative** |  |  |
|  | **75** | **Title:**Biochemical characterization of a lead-tolerant strain of Aspergillus foetidus: An implication of bioremediation of lead from liquid media | **DOI:**10.1016/j.ibiod.2012.05.031 | **oxidative** |  |  |
|  | **76** | **Title:**Autophagy promotes survival in aging submerged cultures of the filamentous fungus Aspergillus niger | **PubMed ID:**23700238 | **oxidative** |  |  |
|  | **77** | **Title:**Genotypic and Phenotypic Versatility of Aspergillus flavus during Maize Exploitation | **PubMed ID:**23894339 | **oxidative** |  |  |
|  | **78** | **Title:**Differences in the Regulation of Ochratoxin A by the HOG Pathway in Penicillium and Aspergillus in Response to High Osmolar Environments | **PubMed ID:**23877195 | **oxidative** | **osmotic** | **CWI** |
|  | **79** | **Title:**RsmA Regulates Aspergillus fumigatus Gliotoxin Cluster Metabolites Including Cyclo(L-Phe-L-Ser), a Potential New Diagnostic Marker for Invasive Aspergillosis | **PubMed ID:**23671611 | **oxidative** |  |  |
|  | **80** | **Title:**Effect of cell wall integrity stress and RlmA transcription factor on asexual development and autolysis in Aspergillus nidulans | **PubMed ID:**23485399 | **oxidative** | **CWI** |  |
|  | **81** | **Title:**Effects of a Defective Endoplasmic Reticulum-Associated Degradation Pathway on the Stress Response, Virulence, and Antifungal Drug Susceptibility of the Mold Pathogen Aspergillus fumigatus | **PubMed ID:**23355008 | **oxidative** | **CWI** |  |
|  | **82** | **Title:**LAMMER Kinase LkhA Plays Multiple Roles in the Vegetative Growth and Asexual and Sexual Development of Aspergillus nidulans | **PubMed ID:**23516554 | **oxidative** |  |  |
|  | **83** | **Title:**Effects of U0126 on growth and activation of mitogen-activated protein kinases in Aspergillus fumigatus | **PubMed ID:**23324267 | **oxidative** |  |  |
|  | **84** | **Title:**bZIP transcription factors affecting secondary metabolism, sexual development and stress responses in Aspergillus nidulans | **PubMed ID:**23154967 | **oxidative** |  |  |
|  | **85** | **Title:**Evaluation for Rock Phosphate Solubilization in Fermentation and Soil-Plant System Using A Stress-Tolerant Phosphate-Solubilizing Aspergillus niger WHAK1 | **PubMed ID:**23229476 | **oxidative** |  |  |
|  | **86** | **Title:**NikA/TcsC Histidine Kinase Is Involved in Conidiation, Hyphal Morphology, and Responses to Osmotic Stress and Antifungal Chemicals in Aspergillus fumigatus | **PubMed ID:**24312504 | **osmotic** | **CWI** |  |
|  | **87** | **Title:**Effect of phenylpyrrole-resistance on fitness parameters and ochratoxin production in Aspergillus carbonarius | **PubMed ID:**23800740 | **osmotic** |  |  |
|  | **88** | **Title:**Role of the Zinc Finger Transcription Factor SltA in Morphogenesis and Sterigmatocystin Biosynthesis in the Fungus Aspergillus nidulans | **PubMed ID:**23840895 | **osmotic** |  |  |
|  | **89** | **Title:**Sensitivity of Aspergillus nidulans to the Cellulose Synthase Inhibitor Dichlobenil: Insights from Wall-Related Genes' Expression and Ultrastructural Hyphal Morphologies | **PubMed ID:**24312197 | **CWI** |  |  |
|  | **90** | **Title:**The Transcriptional Repressor TupA in Aspergillus niger Is Involved in Controlling Gene Expression Related to Cell Wall Biosynthesis, Development, and Nitrogen Source Availability | **PubMed ID:**24205111 | **CWI** |  |  |
|  | **91** | Title: Transcriptomic and morphological profiling of Aspergillus fumigatus Af293 in response to antifungal activity produced byLactobacillus plantarum 16Development, and Nitrogen Source Availability | **PubMed ID:**23876797 | **CWI** |  |  |
|  | **92** | **Title:**Aspergillus oryzae AoSO Is a Novel Component of Stress Granules upon Heat Stress in Filamentous Fungi | **PubMed ID:**23991062 | **CWI** |  |  |
|  | **93** | **Title:**N-Glycosylation of Gel1 or Gel2 is vital for cell wall beta-glucan synthesis in Aspergillus fumigatus | **PubMed ID:**23650256 | **CWI** |  |  |
|  | **94** | **Title:**Transcriptome and Biochemical Analysis Reveals That Suppression of GPI-Anchor Synthesis Leads to Autophagy and Possible Necroptosis in Aspergillus fumigatus | **PubMed ID:**23527074 | **CWI** |  |  |
|  | **95** | **Title:**Functional Analysis of the Aspergillus nidulans Kinome | **PubMed ID:**23505451 | **CWI** |  |  |
|  | **96** | **Title:**Functional Characterization of Aspergillus nidulans ypkA, a Homologue of the Mammalian Kinase SGK | **PubMed ID:**23472095 | **CWI** |  |  |
|  | **97** | **Title:**Role of the Guanine Nucleotide Exchange Factor Rom2 in Cell Wall Integrity Maintenance of Aspergillus fumigatus | **PubMed ID:**23264643 | **CWI** |  |  |
|  | **98** | **Title:**Aspergillus fumigatus melanins: interference with the host endocytosis pathway and impact on virulence | **PubMed ID:**23346079 | **CWI** |  |  |
|  | **99** | **Title:**Laccases Involved in 1,8-Dihydroxynaphthalene Melanin Biosynthesis in Aspergillus fumigatus Are Regulated by Developmental Factors and Copper Homeostasis | **PubMed ID:**24123270 | **other** |  |  |
|  | **100** | **Title:**Investigation of Malic Acid Production in Aspergillus oryzae under Nitrogen Starvation Conditions | **PubMed ID:**23892740 | **other** |  |  |
|  | **101** | **Title:**Comparison of growth, nutritional utilisation patterns, and niche overlap indices of toxigenic and atoxigenic Aspergillus flavus strains | **PubMed ID:**24012304 | **other** |  |  |
|  | **102** | **Title:**Comparison and Analysis of the Genomes of Two Aspergillus oryzae Strains | **PubMed ID:**23889147 | **other** |  |  |
|  | **103** | **Title:**Effect of Kluyveromyces thermotolerans on polyketide synthase gene expression and ochratoxin accumulation by Penicillium and Aspergillus | **DOI:**10.3920/WMJ2012.1532 | **other** |  |  |
|  | **104** | **Title:**Phospho-regulation and nucleocytoplasmic trafficking of CrzA in response to calcium and alkaline-pH stress in Aspergillus nidulans | **PubMed ID:**23772954 | **other** |  |  |
|  | **105** | **Title:**conF and conJ contribute to conidia germination and stress response in the filamentous fungus Aspergillus nidulans | **PubMed ID:**23644150 | **other** |  |  |
|  | **106** | **Title:**Genetic Bypass of Aspergillus nidulans crzA Function in Calcium Homeostasis | **PubMed ID:**23665873 | **other** |  |  |
|  | **107** | **Title:**The Aspergillus nidulans Peripheral ER: Disorganization by ER Stress and Persistence during Mitosis | **PubMed ID:**23826221 | **other** |  |  |
|  | **108** | **Title:**Deletion of the sec4 Homolog srgA from Aspergillus fumigatus Is Associated with an Impaired Stress Response, Attenuated Virulence and Phenotypic Heterogeneity | **PubMed ID:**23785510 | **other** |  |  |
|  | **109** | **Title:**The Spt-Ada-Gcn5 Acetyltransferase (SAGA) Complex in Aspergillus nidulans | **PubMed ID:**23762321 | **other** |  |  |
|  | **110** | **Title:**Molecular Genetic Characterization of the Biosynthesis Cluster of a Prenylated Isoindolinone Alkaloid Aspernidine A in Aspergillus nidulans | **PubMed ID:**23706169 | **other** |  |  |
|  | **111** | **Title:**Pathway of Glycine Betaine Biosynthesis in Aspergillus fumigatus | **PubMed ID:**23563483 | **other** |  |  |
|  | **112** | **Title:**Identification of regulatory elements in the glucoamylase-encoding gene (glaB) promoter from Aspergillus oryzae | **PubMed ID:**23224588 | **other** |  |  |
|  | **113** | **Title:**Transcriptomic Insights into the Oxidative Response of Stress-Exposed Aspergillus fumigatus | **PubMed ID:**23278536 | **other** |  |  |
|  | **114** | **Title:**Identification of Metabolic Pathways Influenced by the G-Protein Coupled in Aspergillus nidulans | **PubMed ID:**23658706 | **other** |  |  |
|  | **115** | **Title:**pyrG is required for maintaining stable cellular uracil level and normal sporulation pattern under excess uracil stress in Aspergillus nidulans | **PubMed ID:**23633078 | **other** |  |  |
|  | **116** | **Title:**Azole resistant Aspergillus fumigatus: An emerging problem | **PubMed ID:**23562488 | **other** |  |  |
|  | **117** | **Title:**Biochemical and Biophysical Response to Calcium Chloride Stress in Aspergillus niger and its Role in Malachite Green Degradation | **PubMed ID:**23076635 | **other** |  |  |
|  | **118** | **Title:**Modelling colony population growth in the filamentous fungus Aspergillus nidulans | **PubMed ID:**23246716 | **other** |  |  |
|  | **119** | **Title:**The Fungal Pathogen Aspergillus fumigatus Regulates Growth, Metabolism, and Stress Resistance in Response to Light | **PubMed ID:**23532976 | **other** |  |  |
|  | **120** | **Title:**Extracellular DNA Release Acts as an Antifungal Resistance Mechanism in Mature Aspergillus fumigatus Biofilms | **PubMed ID:**23314962 | **other** |  |  |
|  | **121** | **Title:**Intracellular trehalase activity is required for development, germination and heat-stress resistance of Aspergillus niger conidia | **PubMed ID:**23116628 | **other** |  |  |
|  | **122** | **Title:**In vitro and in vivo role of heat shock protein 90 in Amphotericin B resistance of Aspergillus terreus | **PubMed ID:**22515428 | **other** |  |  |
| **2012** | **123** | **Title:**Involvement of Protein Kinase C in the Suppression of Apoptosis and in Polarity Establishment in Aspergillus nidulans under Conditions of Heat Stress | **PubMed ID:**23209763 | **oxidative** | **osmotic** | **CWI** |
|  | **124** | **Title:**Characterization of the velvet regulators in Aspergillus fumigatus | **PubMed ID:**22970834 | **oxidative** |  |  |
|  | **125** | **Title:**How Peroxisomes Affect Aflatoxin Biosynthesis in Aspergillus Flavus | **PubMed ID:**23094106 | **oxidative** |  |  |
|  | **126** | **Title:**The Aspergillus fumigatus Protein GliK Protects against Oxidative Stress and Is Essential for Gliotoxin Biosynthesis | **PubMed ID:**22903976 | **oxidative** |  |  |
|  | **127** | **Title:**Aoyap1 regulates OTA synthesis by controlling cell redox balance in Aspergillus ochraceus | **PubMed ID:**22410746 | **oxidative** |  |  |
|  | **128** | **Title:**Genome-wide expression analysis upon constitutive activation of the HacA bZIP transcription factor in Aspergillus niger reveals a coordinated cellular response to counteract ER stress | **PubMed ID:**22846479 | **oxidative** |  |  |
|  | **129** | **Title:**Proteome analysis of the farnesol-induced stress response in Aspergillus nidulans-The role of a putative dehydrin | **PubMed ID:**22634043 | **oxidative** | **osmotic** |  |
|  | **130** | **Title:**GmcA Is a Putative Glucose-Methanol-Choline Oxidoreductase Required for the Induction of Asexual Development in Aspergillus nidulans | **PubMed ID:**22792266 | **oxidative** | **osmotic** |  |
|  | **131** | **Title:**The anisin1 gene encodes a defensin-like protein and supports the fitness of Aspergillus nidulans | **PubMed ID:**22113351 | **oxidative** |  |  |
|  | **132** | **Title:**Development stage-specific proteomic profiling uncovers small, lineage specific proteins most abundant in the Aspergillus Fumigatus conidial proteome | **PubMed ID:**22545825 | **oxidative** | **CWI** |  |
|  | **133** | **Title:**Transcriptional and Proteomic Analysis of the Aspergillus fumigatus Delta prtT Protease-Deficient Mutant | **PubMed ID:**22514608 | **oxidative** |  |  |
|  | **134** | **Title:**Aspergillus fumigatus mitochondrial electron transport chain mediates oxidative stress homeostasis, hypoxia responses and fungal pathogenesis | **PubMed ID:**22443190 | **oxidative** |  |  |
|  | **135** | **Title:**Molecular Characterization of the Putative Transcription Factor SebA Involved in Virulence in Aspergillus fumigatus | **PubMed ID:**22345349 | **oxidative** | **CWI** |  |
|  | **136** | **Title:**Visual expression analysis of the responses of the alternative oxidase gene (aox1) to heat shock, oxidative, and osmotic stresses in conidia of citric acid-producing Aspergillus niger | **PubMed ID:**22138384 | **oxidative** | **CWI** |  |
|  | **137** | **Title:**A novel dehydrin-like protein from Aspergillus fumigatus regulates freezing tolerance | **PubMed ID:**22306917 | **oxidative** | **osmotic** |  |
|  | **138** | **Title:**An Aspergillus nidulans bZIP response pathway hardwired for defensive secondary metabolism operates through aflR | **PubMed ID:**22283524 | **oxidative** |  |  |
|  | **139** | **Title:**Mevalonate governs interdependency of ergosterol and siderophore biosyntheses in the fungal pathogen Aspergillus fumigatus | **PubMed ID:**22106303 | **oxidative** |  |  |
|  | **140** | **Title:**Heme-Biosynthetic Porphobilinogen Deaminase Protects Aspergillus nidulans from Nitrosative Stress | **PubMed ID:**22038601 | **oxidative** |  |  |
|  | **141** | **Title:**Putative Calcium Channels CchA and MidA Play the Important Roles in Conidiation, Hyphal Polarity and Cell Wall Components in Aspergillus nidulans | **PubMed ID:**23071589 | **osmotic** | **CWI** |  |
|  | **142** | **Title:**Microcolony Imaging of Aspergillus fumigatus Treated with Echinocandins Reveals Both Fungistatic and Fungicidal Activities | **PubMed ID:**22536390 | **osmotic** | **CWI** |  |
|  | **143** | **Title:**Vacuolar H+-ATPase plays a key role in cell wall biosynthesis of Aspergillus niger | **PubMed ID:**22222772 | **osmotic** | **CWI** |  |
|  | **144** | **Title:**Heat Shock Protein 90 Is Required for Conidiation and Cell Wall Integrity in Aspergillus fumigatus | **PubMed ID:**22822234 | **CWI** |  |  |
|  | **145** | **Title:**HOG-MAPK Signaling Regulates the Adaptive Responses of Aspergillus fumigatus to Thermal Stress and Other Related Stress | **PubMed ID:**22678624 | **CWI** |  |  |
|  | **146** | **Title:**The echinocandin B producer fungus Aspergillus nidulans var. roseus ATCC 58397 does not possess innate resistance against its lipopeptide antimycotic | **PubMed ID:**22555909 | **CWI** |  |  |
|  | **147** | **Title:**Deciphering cell wall integrity signalling in Aspergillus fumigatus: identification and functional characterization of cell wall stress sensors and relevant Rho GTPases | **PubMed ID:**22220813 | **CWI** |  |  |
|  | **148** | **Title:**The transcriptomic fingerprint of glucoamylase over-expression in Aspergillus niger | **PubMed ID:**23237452 | **other** |  |  |
|  | **149** | **Title:**Dsc Orthologs Are Required for Hypoxia Adaptation, Triazole Drug Responses, and Fungal Virulence in Aspergillus fumigatus | **PubMed ID:**23104569 | **other** |  |  |
|  | **150** | **Title:**NosA, a transcription factor important in Aspergillus fumigatus stress and developmental response, rescues the germination defect of a laeA deletion | **PubMed ID:**23022264 | **other** |  |  |
|  | **151** | **Title:**The Role, Interaction and Regulation of the Velvet Regulator VelB in Aspergillus nidulans | **PubMed ID:**23049895 | **other** |  |  |
|  | **152** | **Title:**Aspergillus fumigatus calcineurin interacts with a nucleoside diphosphate kinase | **PubMed ID:**22634424 | **other** |  |  |
|  | **153** | **Title:**The Two-Component Sensor Kinase TcsC and Its Role in Stress Resistance of the Human-Pathogenic Mold Aspergillus fumigatus | **PubMed ID:**22675534 | **other** |  |  |
|  | **154** | **Title:**HOG-MAPK signaling regulates the adaptive responses of Aspergillus fumigatus to environmental stresses | **ISSN:**0933-7407 | **other** |  |  |
|  | **155** | **Title:**Effect of a Cultures of Aspergillus oryzae on Inflammatory Response and mRNA Expression in Intestinal Immune-Related Mediators of Male Broiler Chicks | **DOI:**10.2141/jpsa.011079 | **other** |  |  |
|  | **156** | **Title:**Influence of sub-lethal antioxidant doses, water potential and temperature on growth, sclerotia, aflatoxins and aflD (=nor-1) expression by Aspergillus flavus RCP08108 | **PubMed ID:**22227105 | **other** |  |  |
| **2011** | **157** | **Title:**The Role of sho1 in Polarized Growth of Aspergillus fumigatus | **PubMed ID:**21796487 | **oxidative** | **osmotic** | **CWI** |
|  | **158** | **Title:**A novel family of dehydrin-like proteins is involved in stress response in the human fungal pathogen Aspergillus fumigatus | **PubMed ID:**21490150 | **oxidative** | **osmotic** |  |
|  | **159** | **Title:**Putative Stress Sensors WscA and WscB Are Involved in Hypo-Osmotic and Acidic pH Stress Tolerance in Aspergillus nidulans | **PubMed ID:**21926329 | **oxidative** | **CWI** |  |
|  | **160** | **Title:**The metalloreductase FreB is involved in adaptation of Aspergillus fumigatus to iron starvation | **PubMed ID:**21840411 | **oxidative** |  |  |
|  | **161** | **Title:**Osmotic stabilizer-coupled suppression of NDR defects is dependent on the calcium-calcineurin signaling cascade in Aspergillus nidulans | **PubMed ID:**21741477 | **oxidative** | **osmotic** |  |
|  | **162** | **Title:**The MAP kinase MpkA controls cell wall integrity, oxidative stress response, gliotoxin production and iron adaptation in Aspergillus fumigatus | **PubMed ID:**21883519 | **oxidative** | **CWI** |  |
|  | **163** | **Title:**Targeted Disruption of Nonribosomal Peptide Synthetase pes3 Augments the Virulence of Aspergillus fumigatus | **PubMed ID:**21746855 | **oxidative** |  |  |
|  | **164** | **Title:**Asperlin From the Marine-Derived Fungus Aspergillus sp SF-5044 Exerts Anti-inflammatory Effects Through Heme Oxygenase-1 Expression in Murine Macrophages | **PubMed ID:**21705844 | **oxidative** |  |  |
|  | **165** | **Title:**Repression of N-glycosylation triggers the unfolded protein response (UPR) and overexpression of cell wall protein and chitin in Aspergillus fumigatus | **PubMed ID:**21527474 | **oxidative** | **CWI** |  |
|  | **166** | **Title:**SidL, an Aspergillus fumigatus Transacetylase Involved in Biosynthesis of the Siderophores Ferricrocin and Hydroxyferricrocin | **PubMed ID:**21622789 | **oxidative** |  |  |
|  | **167** | **Title:**Aspergillus nidulans transcription factor AtfA interacts with the MAPK SakA to regulate general stress responses, development and spore functions | **PubMed ID:**21320182 | **oxidative** | **osmotic** |  |
|  | **168** | **Title:**Characterization of the conserved phosphorylation site in the Aspergillus nidulans response regulator SrrA | **PubMed ID:**21229249 | **oxidative** | **osmotic** | **CWI** |
|  | **169** | **Title:**Elucidation of Functional Markers from Aspergillus nidulans Developmental Regulator FlbB and Their Phylogenetic Distribution | **PubMed ID:**21423749 | **oxidative** |  |  |
|  | **170** | **Title:**Loss of msnA, a Putative Stress Regulatory Gene, in Aspergillus parasiticus and Aspergillus flavus Increased Production of Conidia, Aflatoxins and Kojic Acid | **PubMed ID:**22069691 | **oxidative** |  |  |
|  | **171** | **Title:**Morphology engineering - Osmolality and its effect on Aspergillus niger morphology and productivity | **PubMed ID:**21801352 | **osmotic** | **CWI** |  |
|  | **172** | **Title:**Analysis of the Aspergillus fumigatus Proteome Reveals Metabolic Changes and the Activation of the Pseurotin A Biosynthesis Gene Cluster in Response to Hypoxia | **PubMed ID:**21388144 | **osmotic** |  |  |
|  | **173** | **Title:**8-Carbon oxylipins inhibit germination and growth, and stimulate aerial conidiation in Aspergillus nidulans | **PubMed ID:**21530921 | **osmotic** |  |  |
|  | **174** | **Title:**Impact of a Streptomyces (AS1) strain and its metabolites on control of Aspergillus flavus and aflatoxin B-1 contamination in vitro and in stored peanuts | **DOI:**10.1080/09583157.2011.632078 | **osmotic** |  |  |
|  | **175** | **Title:**The virulence of the opportunistic fungal pathogen Aspergillus fumigatus requires cooperation between the endoplasmic reticulum-associated degradation pathway (ERAD) and the unfolded protein response (UPR) | **PubMed ID:**21217201 | **osmotic** |  |  |
|  | **176** | **Title:**Localization and activity of the calcineurin catalytic and regulatory subunit complex at the septum is essential for hyphal elongation and proper septation in Aspergillus fumigatus | **PubMed ID:**22066998 | **CWI** |  |  |
|  | **177** | **Title:**HacA-Independent Functions of the ER Stress Sensor IreA Synergize with the Canonical UPR to Influence Virulence Traits in Aspergillus fumigatus | **PubMed ID:**22028661 | **CWI** |  |  |
|  | **178** | **Title:**The chitin synthase genes chsA and chsC are not required for cell wall stress responses in the human pathogen Aspergillus fumigatus | **PubMed ID:**21763289 | **CWI** |  |  |
|  | **179** | **Title:**beta-1,3-Glucan-Induced Host Phospholipase D Activation Is Involved in Aspergillus fumigatus Internalization into Type II Human Pneumocyte A549 Cells | **PubMed ID:**21760893 | **CWI** |  |  |
|  | **180** | **Title:**AbaA and WetA govern distinct stages of Aspergillus fumigatus development | **PubMed ID:**20966095 | **CWI** |  |  |
|  | **181** | **Title:**Divergent Protein Kinase A isoforms co-ordinately regulate conidial germination, carbohydrate metabolism and virulence in Aspergillus fumigatus | **PubMed ID:**21210869 | **CWI** |  |  |
|  | **182** | **Title:**Interrogation of Related Clinical Pan-Azole-Resistant Aspergillus fumigatus Strains: G138C, Y431C, and G434C Single Nucleotide Polymorphisms in cyp51A, Upregulation of cyp51A, and Integration and Activation of Transposon Atf1 in the cyp51A Promoter | **PubMed ID:**21876055 | **CWI** |  |  |
|  | **183** | **Title:**Impact of the Lectin Chaperone Calnexin on the Stress Response, Virulence and Proteolytic Secretome of the Fungal Pathogen Aspergillus fumigatus | **PubMed ID:**22163332 | **other** |  |  |
|  | **184** | **Title:**Isolation and characterization of self-fertile suppressors from the sterile nsdD deletion mutant of Aspergillus nidulans | **PubMed ID:**22203574 | **other** |  |  |
|  | **185** | **Title:**Berberine and Itraconazole Are not Synergistic in Vitro against Aspergillus fumigatus Isolated from Clinical Patients | **DOI:**10.3390/molecules16119218 | **other** |  |  |
|  | **186** | **Title:**Role of Nitric Oxide and Flavohemoglobin Homolog Genes in Aspergillus nidulans Sexual Development and Mycotoxin Production | **PubMed ID:**21642398 | **other** |  |  |
|  | **187** | **Title:**The MpkB MAP Kinase Plays a Role in Post-karyogamy Processes as well as in Hyphal Anastomosis During Sexual Development in Aspergillus nidulans | **PubMed ID:**21717328 | **other** |  |  |
|  | **188** | **Title:**Functional analysis and subcellular location of two flavohemoglobins from Aspergillus oryzae | **PubMed ID:**20817113 | **other** |  |  |
|  | **189** | **Title:**Interplanetary survival probability of Aspergillus terreus spores under simulated solar vacuum ultraviolet irradiation | **DOI:**10.1016/j.pss.2010.11.002 | **other** |  |  |
| **2010** | **190** | **Title:**Approaching the Secrets of N-Glycosylation in Aspergillus fumigatus: Characterization of the AfOch1 Protein | **PubMed ID:**21206755 | **oxidative** | **CWI** |  |
|  | **191** | **Title:**Involvement of the Aspergillus nidulans protein kinase C with farnesol tolerance is related to the unfolded protein response | **PubMed ID:**21091509 | **oxidative** | **CWI** |  |
|  | **192** | **Title:**Augmenting the activity of antifungal agents against aspergilli using structural analogues of benzoic acid as chemosensitizing agents | **PubMed ID:**20943191 | **oxidative** |  |  |
|  | **193** | **Title:**Truncated Afyap1 Attenuates Antifungal Susceptibility of Aspergillus fumigatus to Voriconazole and Confers Adaptation of the Fungus to Oxidative Stress | **PubMed ID:**20376564 | **oxidative** |  |  |
|  | **194** | **Title:**Role of Trehalose Biosynthesis in Aspergillus fumigatus Development, Stress Response, and Virulence | **PubMed ID:**20439478 | **oxidative** | **CWI** |  |
|  | **195** | **Title:**Heptahelical Receptors GprC and GprD of Aspergillus fumigatus Are Essential Regulators of Colony Growth, Hyphal Morphogenesis, and Virulence | **PubMed ID:**20418440 | **oxidative** |  |  |
|  | **196** | **Title:**Novel insights into the functional role of three protein arginine methyltransferases in Aspergillus nidulans | **PubMed ID:**20338257 | **oxidative** |  |  |
|  | **197** | **Title:**DNA Damage and DNA Damage Responses in THP-1 Monocytes after Exposure to Spores of either Stachybotrys chartarum or Aspergillus versicolor or to T-2 toxin | **PubMed ID:**20150440 | **oxidative** |  |  |
|  | **198** | **Title:**Aspergillus oryzae flavohemoglobins promote oxidative damage by hydrogen peroxide | **PubMed ID:**20211603 | **oxidative** |  |  |
|  | **199** | **Title:**Distinct enzymatic and cellular characteristics of two secretory phospholipases A(2) in the filamentous fungus Aspergillus oryzae | **PubMed ID:**20045482 | **oxidative** |  |  |
|  | **200** | **Title:**AtfA bZIP-type transcription factor regulates oxidative and osmotic stress responses in Aspergillus nidulans | **PubMed ID:**20131067 | **oxidative** | **osmotic** |  |
|  | **201** | **Title:**Beyond aflatoxin: four distinct expression patterns and functional roles associated with Aspergillus flavus secondary metabolism gene clusters | **PubMed ID:**20447271 | **oxidative** |  |  |
|  | **202** | **Title:**Tolerance of arsenate-induced stress in Aspergillus niger, a possible candidate for bioremediation | **PubMed ID:**19811831 | **oxidative** | **heavy metal** |  |
|  | **203** | **Title:**Phylogeny of fungal hemoglobins and expression analysis of the Aspergillus oryzae flavohemoglobin gene fhbA during hyphal growth | **PubMed ID:**20960969 | **oxidative** |  |  |
|  | **204** | **Title:**Functional analysis of the superoxide dismutase family in Aspergillus fumigatus | **PubMed ID:**20487287 | **oxidative** |  |  |
|  | **205** | **Title:**Use of the Aspergillus oryzae actin gene promoter in a novel reporter system for exploring antifungal compounds and their target genes | **PubMed ID:**20464390 | **osmotic** |  |  |
|  | **206** | **Title:**Modelling growth of Penicillium expansum and Aspergillus niger at constant and fluctuating temperature conditions | **PubMed ID:**20413170 | **osmotic** |  |  |
|  | **207** | **Title:**The relationship between cellular and calcium responses of Aspergillus awamori to external influences | **DOI:**10.1134/S0026261710030033 | **osmotic** |  |  |
|  | **208** | **Title:**AfMkk2 is required for cell wall integrity signaling, adhesion, and full virulence of the human pathogen Aspergillus fumigatus | **PubMed ID:**20452278 | **CWI** |  |  |
|  | **209** | **Title:**Transcriptional Regulation of Chitin Synthases by Calcineurin Controls Paradoxical Growth of Aspergillus fumigatus in Response to Caspofungin | **PubMed ID:**20124000 | **CWI** |  |  |
|  | **210** | **Title:**The role of thiol species in the tolerance of Aspergillus niger b77 to cadmium ions | **ISSN:**0209-4541 | **heavy metal** |  |  |
|  | **211** | **Title:**Transcriptome analysis of nitrate assimilation in Aspergillus nidulans reveals connections to nitric oxide metabolism | **PubMed ID:**20969648 | **other** |  |  |
|  | **212** | **Title:**Spatial and Developmental Differentiation of Mannitol Dehydrogenase and Mannitol-1-Phosphate Dehydrogenase in Aspergillus niger | **PubMed ID:**20305000 | **other** |  |  |
|  | **213** | **Title:**Identification of possible targets of the Aspergillus fumigatus CRZ1 homologue, CrzA | **PubMed ID:**20078882 | **other** |  |  |
|  | **214** | **Title:**A Novel Motif in Fungal Class 1 Histone Deacetylases Is Essential for Growth and Development of Aspergillus | **PubMed ID:**19940017 | **other** |  |  |
|  | **215** | **Title:**AoSO protein accumulates at the septal pore in response to various stresses in the filamentous fungus Aspergillus oryzae | **PubMed ID:**19945422 | **other** |  |  |
|  | **216** | **Title:**Morphology of Filamentous Fungi: Linking Cellular Biology to Process Engineering Using Aspergillus niger | **PubMed ID:**20490972 | **other** |  |  |
|  | **217** | **Title:**Mutagenesis of Aspergillus oryzae ipt-301 to improve the production of beta-fructofuranosidase | **PubMed ID:**24031480 | **other** |  |  |
| **2009** | **218** | **Title:**Proteome analysis of Aspergillus niger: Lactate added in starch-containing medium can increase production of the mycotoxin fumonisin B-2 by modifying acetyl-CoA metabolism | **PubMed ID:**20003296 | **oxidative** |  |  |
|  | **219** | **Title:**Aspergillus oryzae atfA controls conidial germination and stress tolerance | **PubMed ID:**19770065 | **oxidative** |  |  |
|  | **220** | **Title:**Effect of ozone on spore germination, spore production and biomass production in two Aspergillus species | **PubMed ID:**19533409 | **oxidative** |  |  |
|  | **221** | **Title:**Transcriptional profiling for Aspergillus nidulans HogA MAPK signaling pathway in response to fludioxonil and osmotic stress | **PubMed ID:**19596074 | **oxidative** | **osmotic** |  |
|  | **222** | **Title:**HdaA, a class 2 histone deacetylase of Aspergillus fumigatus, affects germination and secondary metabolite production | **PubMed ID:**19563902 | **oxidative** |  |  |
|  | **223** | **Title:**Phenotypic analysis of genes whose mRNA accumulation is dependent on calcineurin in Aspergillus fumigatus | **PubMed ID:**19573616 | **oxidative** |  |  |
|  | **224** | **Title:**Crawler, a novel Tc1/mariner-type transposable element in Aspergillus oryzae transposes under stress conditions | **PubMed ID:**19269345 | **oxidative** |  |  |
|  | **225** | **Title:**The Glutathione System of Aspergillus nidulans Involves a Fungus-specific Glutathione S-Transferase | **PubMed ID:**19171936 | **oxidative** |  |  |
|  | **226** | **Title:**Effect of natural maize phytochemicals on Aspergillus section Flavi sclerotia characteristics under different conditions of growth media and water potential | **DOI:**10.1016/j.funeco.2008.10.004 | **oxidative** |  |  |
|  | **227** | **Title:**A Role for the Unfolded Protein Response (UPR) in Virulence and Antifungal Susceptibility in Aspergillus fumigatus | **PubMed ID:**19132084 | **oxidative** | **CWI** |  |
|  | **228** | **Title:**Characterization of NikA Histidine Kinase and Two Response Regulators with Special Reference to Osmotic Adaptation and Asexual Development in Aspergillus nidulans | **PubMed ID:**19584543 | **osmotic** |  |  |
|  | **229** | **Title:**The MpkA MAP kinase module regulates cell wall integrity signaling and pyomelanin formation in Aspergillus fumigatus | **PubMed ID:**19715768 | **CWI** |  |  |
|  | **230** | **Title:**Alternative Processing of Proproteins in Aspergilli kexB Gene Disruptants under Hyperosmotic Conditions | **PubMed ID:**19129662 | **CWI** |  |  |
|  | **231** | **Title:**Kapl, a non-essential member of the Pse1p/Imp5 karyopherin family, controls colonial and asexual development in Aspergillus nidulans | **PubMed ID:**19729403 | **other** |  |  |
|  | **232** | **Title:**The bZIP-type transcription factor FlbB regulates distinct morphogenetic stages of colony formation in Aspergillus nidulans | **PubMed ID:**19656299 | **other** |  |  |
|  | **233** | **Title:**The nsdC Gene Encoding a Putative C2H2-Type Transcription Factor Is a Key Activator of Sexual Development in Aspergillus nidulans | **PubMed ID:**19416940 | **other** |  |  |
|  | **234** | **Title:**Aspergillus fumigatus Calcipressin CbpA Is Involved in Hyphal Growth and Calcium Homeostasis | **PubMed ID:**19252123 | **other** |  |  |
|  | **235** | **Title:**Aspergillus fumigatus metabolism: Clues to mechanisms of in vivo fungal growth and virulence | **PubMed ID:**19253141 | **other** |  |  |
|  | **236** | **Title:**Aspergillus nidulans FlbE is an upstream developmental activator of conidiation functionally associated with the putative transcription factor FlbB | **PubMed ID:**19007409 | **other** |  |  |
| **2008** | **237** | **Title:**Silencing of mitochondrial alternative oxidase gene of Aspergillus fumigatus enhances reactive oxygen species production and killing of the fungus by macrophages | **PubMed ID:**19148712 | **oxidative** |  |  |
|  | **238** | Title: Characterization of bZip-Type Transcription Factor AtfA with Reference to Stress Responses of Conidia of Aspergillus nidulans | **PubMed ID:**18838792 | **oxidative** |  |  |
|  | **239** | **Title:**Aspergillus parasiticus crzA, Which Encodes Calcineurin Response Zinc-Finger Protein, Is Required for Aflatoxin Production under Calcium Stress | **PubMed ID:**19325734 | **oxidative** | **osmotic** |  |
|  | **240** | Title: SreA-mediated iron regulation in Aspergillus fumigatus | **PubMed ID:**18721228 | **oxidative** |  |  |
|  | **241** | **Title:**Regulation of apical dominance in Aspergillus nidulans hyphae by reactive oxygen species | **PubMed ID:**18689883 | **oxidative** |  |  |
|  | **242** | **Title:**Chemosensitization prevents tolerance of Aspergillus fumigatus to antimycotic drugs | **PubMed ID:**18486603 | **oxidative** | **osmotic** | **CWI** |
|  | **243** | **Title:**Defects in conidiophore development and conidium-macrophage interactions in a dioxygenase mutant of Aspergillus fumigatus | **PubMed ID:**18443090 | **oxidative** |  |  |
|  | **244** | **Title:**Modulation of antioxidant defense in Aspergillus parasiticus is involved in aflatoxin biosynthesis: a role for the ApyapA gene | **PubMed ID:**18441122 | **oxidative** |  |  |
|  | **245** | Title: Aspergillus oryzae atfB encodes a transcription factor required for stress tolerance in conidia | **PubMed ID:**18448366 | **oxidative** | **osmotic** |  |
|  | **246** | **Title:**The mitogen-activated protein kinase MpkA of Aspergillus fumigatus regulates cell wall signaling and oxidative stress response | **PubMed ID:**17981060 | **oxidative** | **CWI** |  |
|  | **247** | **Title:**The sho1 sensor regulates growth, morphology, and oxidant adaptation in Aspergillus fumigatus but is not essential for development of invasive pulmonary aspergillosis | **PubMed ID:**18227163 | **oxidative** |  |  |
|  | **248** | **Title:**Functional characterization of the putative Aspergillus nidulans DNA damage binding protein homologue DdbA | **PubMed ID:**18060432 | **oxidative** |  |  |
|  | **249** | **Title:**A novel screening method for cell wall mutants in Aspergillus niger identifies UDP-galactopyranose mutase as an important protein in fungal cell wall biosynthesis | **PubMed ID:**18245853 | **oxidative** | **CWI** |  |
|  | **250** | **Title:**Cd (II) stress response during the growth of Aspergillus niger B 77 | **PubMed ID:**17850314 | **oxidative** | **heavy metal** |  |
|  | **251** | **Title:**Afyap1, encoding a bZip transcriptional factor of Aspergillus fumigatus, contributes to oxidative stress response but is not essential to the virulence of this pathogen in mice immunosuppressed by cyclophosphamide and triamcinolone | **PubMed ID:**18608886 | **oxidative** |  |  |
|  | **252** | **Title:**Depletion of the MobB and CotA complex in Aspergillus nidulans causes defects in polarity maintenance that can be suppressed by the environment stress | **PubMed ID:**18832040 | **osmotic** |  |  |
|  | **253** | **Title:**A defect of ligD (human lig4 homolog) for nonhomologous end joining significantly improves efficiency of gene-targeting in Aspergillus oryzae | **PubMed ID:**18282727 | **osmotic** |  |  |
|  | **254** | **Title:**Functional analysis of C(2)H(2) zinc finger transcription factor CrzA involved in calcium signaling in Aspergillus nidulans | **PubMed ID:**19002465 | **CWI** |  |  |
|  | **255** | **Title:**Two zinc finger transcription factors, CrzA and SltA, are involved in cation homoeostasis and detoxification in Aspergillus nidulans | **PubMed ID:**18471095 | **CWI** |  |  |
|  | **256** | **Title:**Calcineurin target CrzA regulates conidial germination, hyphal growth, and pathogenesis of Aspergillus fumigatus | **PubMed ID:**18456861 | **CWI** |  |  |
|  | **257** | **Title:**A Comparison of the Unfolded Protein Response in Solid-State with Submerged Cultures of Aspergillus oryzae | **PubMed ID:**18997417 | **other** |  |  |
|  | **258** | **Title:**Calcineurin localizes to the hyphal septum in Aspergillus fumigatus: Implications for septum formation and conidiophore development | **PubMed ID:**18606829 | **other** |  |  |
|  | **259** | **Title:**Adaptive Melanin Response of the Soil Fungus Aspergillus niger to UV Radiation Stress at "Evolution Canyon'', Mount Carmel, Israel | **PubMed ID:**18714346 | **other** |  |  |
|  | **260** | **Title:**Protein kinase a regulates growth, sporulation, and pigment formation in Aspergillus fumigatus | **PubMed ID:**18539819 | **other** |  |  |
|  | **261** | **Title:**Effect of solute and matric potential on in vitro growth and sporulation of strains from a new population of Aspergillus flavus isolated in Italy | **DOI:**10.1016/j.funeco.2008.07.001 | **other** |  |  |
|  | **262** | **Title:**Impaired ribosome biogenesis disrupts the integration between morphogenesis and nuclear duplication during the germination of Aspergillus fumigatus | **PubMed ID:**18296619 | **other** |  |  |
|  | **263** | **Title:**Variation in mycelial growth and morphology of Aspergillus niger with changes in shear stress | **ISSN: 0973-6263** | **other** |  |  |
| **2007** | **264** | **Title:**The Aspergillus fumigatus transcriptional regulator AfYap1 represents the major regulator for defense against reactive oxygen intermediates but is dispensable for pathogenicity in an intranasal mouse infection model | **PubMed ID:**17921349 | **oxidative** |  |  |
|  | **265** | **Title:**Arginine catabolism in Aspergillus nidulans is regulated by the rrmA gene coding for the RNA-binding protein | **PubMed ID:**17719249 | **oxidative** |  |  |
|  | **266** | **Title:**Inhibition of ochratoxin - A production and growth of Aspergillus species by phenolic antioxidant compounds | **PubMed ID:**17874203 | **oxidative** |  |  |
|  | **267** | **Title:**A Novel Regulator Couples Sporogenesis and Trehalose Biogenesis in Aspergillus nidulans | **PubMed ID:**17912349 | **oxidative** |  |  |
|  | **268** | **Title:**Transcriptome analysis of the Aspergillus nidulans AtmA (ATM, Ataxia-Telanglectasia mutated) null mutant | **PubMed ID:**17880424 | **oxidative** |  |  |
|  | **269** | **Title:**The thioredoxin system of the filamentous fungus Aspergillus nidulans - Impact on development and oxidative stress response | **PubMed ID:**17631497 | **oxidative** |  |  |
|  | **270** | **Title:**Response regulators SrrA and SskA are central components of a phosphorelay system involved in stress signal transduction and asexual sporulation in Aspergillus nidulans | **PubMed ID:**17630329 | **oxidative** | **osmotic** | **CWI** |
|  | **271** | **Title:**Novel reporter gene expression systems for monitoring activation of the Aspergillus nidulans HOG pathway | **PubMed ID:**17617716 | **oxidative** | **osmotic** |  |
|  | **272** | **Title:**Characterization of the bZip-type transcription factor NapA with reference a to oxidative stress response in Aspergillus nidulans | **PubMed ID:**17617701 | **oxidative** |  |  |
|  | **273** | **Title:**Characterization of the SKN7 ortholog of Aspergillus fumigatus | **PubMed ID:**17337219 | **oxidative** |  |  |
|  | **274** | **Title:**The SskA and SrrA response regulators are implicated in oxidative stress responses of hyphae and asexual spores in the phosphorelay signaling network of Aspergillus nidulans | **PubMed ID:**17420584 | **oxidative** |  |  |
|  | **275** | **Title:**MpkA-dependent and -independent cell wall integrity signaling in Aspergillus nidulans | **PubMed ID:**17601879 | **osmotic** | **CWI** |  |
|  | **276** | **Title:**Unexpected link between metal ion deficiency and autophagy in Aspergillus fumigatus | **PubMed ID:**17921348 | **heavy metal** |  |  |
|  | **277** | **Title:**Influence of water activity, pH, and temperature on growth of Aspergillus penicillioides and A. terreus, isolated from dry and salted skipjack tuna (Katsuwonus pelamis) meat. | **ISSN:**0798-2259 | **other** |  |  |
|  | **278** | **Title:**The Aspergillus fumigatus metacaspases CasA and CasB facilitate growth under conditions of endoplasmic reticulum stress | **PubMed ID:**17176258 | **other** |  |  |
| **2006** | **279** | **Title:**Novel mitogen-activated protein kinase MpkC of Aspergillus fumigatus is required for utilization of polyalcohol sugars | **PubMed ID:**16998074 | **oxidative** | **osmotic** |  |
|  | **280** | **Title:**Transcriptome analysis of Aspergillus nidulans exposed to camptothecin-induced DNA damage | **PubMed ID:**17030995 | **oxidative** |  |  |
|  | **281** | **Title:**A nonribosomal peptide synthetase (Pes1) confers protection against oxidative stress in Aspergillus fumigatus | **PubMed ID:**16759234 | **oxidative** |  |  |
|  | **282** | **Title:**Effects of mutations in the GanB/RgsA G protein mediated signalling on the autolysis of Aspergillus nidulans | **PubMed ID:**17139616 | **oxidative** |  |  |
|  | **283** | **Title:**Characterisation of the laccase-encoding gene abr2 of the dihydroxynaphthalene-like melanin gene cluster of Aspergillus fumigatus | **PubMed ID:**16988817 | **other** |  |  |
|  | **284** | **Title:**Aspergillus fumigatus induces innate immune responses in alveolar macrophages through the MAPK pathway independently of TLR2 and TLR4 | **PubMed ID:**16951362 | **other** |  |  |
|  | **285** | **Title:**Targeting antioxidative signal transduction and stress response system: control of pathogenic Aspergillus with phenolics that inhibit mitochondrial function | **PubMed ID:**16834605 | **other** |  |  |
| **2005** | **286** | **Title:**Comparison of gene expression signatures of diamide, H2O2 and menadione exposed Aspergillus nidulans cultures - linking genome-wide transcriptional changes to cellular physiology | **PubMed ID:**16368011 | **oxidative** |  |  |
|  | **287** | **Title:**HdaA, a major class 2 histone deacetylase of Aspergillus nidulans, affects growth under conditions of oxidative stress | **PubMed ID:**16215180 | **oxidative** |  |  |
|  | **288** | **Title:**Examination of fungal stress response genes using Saccharomyces cerevisiae as a model system: targeting genes affecting aflatoxin biosynthesis by Aspergillus flavus Link | **PubMed ID:**15614562 | **oxidative** |  |  |
|  | **289** | **Title:**Aspergillus nidulans HOG pathway is activated only by two-component signalling pathway in response to osmotic stress | **PubMed ID:**15882418 | **oxidative** | **osmotic** |  |
|  | **290** | **Title:**Role of antioxidant enzymes in survival of conidiospores of Aspergillus niger 26 under conditions of temperature stress | **PubMed ID:**16162242 | **oxidative** |  |  |
|  | **291** | **Title:**The class V chitin synthase gene csmA is crucial for the growth of the chsA chsC double mutant in Aspergillus nidulans | **PubMed ID:**15665472 | **osmotic** | **CWI** |  |
|  | **292** | **Title:**Expression of agsA, one of five 1,3-alpha-D-glucan synthase-encoding genes in Aspergillus niger, is induced in response to cell wall stress | **PubMed ID:**15670714 | **CWI** |  |  |
|  | **293** | **Title:**Screening of growth- or development-related genes by using genomic library with inducible promoter in Aspergillus nidulans | **PubMed ID:**16410769 | **other** |  |  |
|  | **294** | **Title:**In vitro selection of maize rhizobacteria to study potential biological control of Aspergillus section Flavi and aflatoxin production | **DOI:**10.1007/s10658-005-5548-3 | **other** |  |  |
|  | **295** | **Title:**Comparing artificial and natural selection in rate of adaptation to genetic stress in Aspergillus nidulans | **PubMed ID:**16033548 | **other** |  |  |
|  | **296** | **Title:**Effect of different postharvest drying temperatures on Aspergillus flavus survival and aflatoxin content in five maize hybrids | **PubMed ID:**16013400 | **other** |  |  |
|  | **297** | **Title:**Effect of undecanoic acid on the production of esterases and lipases by Aspergillus nidulans | **ISSN:**1590-4261 | **other** |  |  |
|  | **298** | **Title:**Aspergillus hyphae in infected tissue: Evidence of physiologic adaptation and effect on culture recovery | **PubMed ID:**15634998 | **other** |  |  |
